# Structural, electronic and vibrational properties of few-layer 2H- and 1T-TaSe_2_

**DOI:** 10.1038/srep16646

**Published:** 2015-11-16

**Authors:** Jia-An Yan, Mack A. Dela Cruz, Brandon Cook, Kalman Varga

**Affiliations:** 1 Department of Physics, Astronomy, and Geosciences, Towson University, 8000 York Road, Towson, Md 21252, USA; 2 Center for Nanophase Materials Sciences, Oak Ridge National Laboratory, P.O. Box 2008, Oak Ridge, TN, 37831 USA; 3 Department of Physics and Astronomy, Vanderbilt University, Nashville, Tennessee 37235, USA

## Abstract

Two-dimensional metallic transition metal dichalcogenides (TMDs) are of interest for studying phenomena such as charge-density wave (CDW) and superconductivity. Few-layer tantalum diselenides (TaSe_2_) are typical metallic TMDs exhibiting rich CDW phase transitions. However, a description of the structural, electronic and vibrational properties for different crystal phases and stacking configurations, essential for interpretation of experiments, is lacking. We present first- principles calculations of structural phase energetics, band dispersion near the Fermi level, phonon properties and vibrational modes at the Brillouin zone center for different layer numbers, crystal phases and stacking geometries. Evolution of the Fermi surfaces as well as the phonon dispersions as a function of layer number reveals dramatic dimensionality effects in this CDW material. Our results indicate strong electronic interlayer coupling, detail energetically possible stacking geometries, and provide a basis for interpretation of Raman spectra.

Two-dimensional (2D) nanosheets of layered transition metal dichalcogenides (TMDs) with chemical formula MX_2_ (where M = Mo, W, Nb, Ta, or Ti and X = S, Se, or Te) have received remarkable attention due to their diverse properties and potential applications^1^. The electronic properties of these 2D nanocrystals range from insulating to semiconducting, metallic and even superconducting, and can differ dramatically from bulk crystals^1^. The versatility of these materials is shown by the wide range of reported applications including: electrocatalysts for hydrogen evolution^1^, opto- and spintronics^2^, electrodes and interconnects^3^,^4^, and electro-optical switch and data storage devices^5^.

Early research into metallic TMDs was done with bulk materials and focused on charge-density wave (CDW), a structural distortion which results in a further electronic stabilization of the system, similar to the Peierls distortion in one-dimensional atomic chains^6^,^7^,^8^,^9^. The driving mechanisms for the CDW transition are still not completely understood. Previous studies have mainly discussed the importance of Fermi-surface nesting^6^, van Hove singularities^10^, and electron-phonon coupling^11^,^12^,^13^.

TaSe_2_ is a typical TMD that exhibits rich CDW phase transitions. The bulk 1T-TaSe_2_ transitions from normal state to incommensurate CDW state below 600 K and into commensurate CDW state below 473 K^6^,^7^,^9^. In contrast, the 2H polytype does not transition into the incommensurate CDW state until ~120 K, and the commensurate CDW state starts below 90 K^7^. Besides being an importan CDW material^14^,^15^,^16^,^17^,^18^,^19^,^20^,^21^,^22^,^23^,^24^, TaSe_2_ has interesting applications as interconnect in devices^3^,^4^. The recently reported mechanical exfoliation of few-layer TaSe_2_ (down to four trilayers) leads to a major question: what is the role of dimensionality and interlayer interactions in the evolution of structural and electronic properties including the CDW transitions^25^,^26^,^27^,^28^,^29^,^30^,^31^. Recent experiments have already demonstrated that the CDW transition temperature of mechanically exfoliated TiSe_2_ films increases from ~200 K^32^ to ~240 K^33^ after the thickness is reduced to a few nanometers. Similar effect has also been reported for ultrathin nanosheets of VSe_2_ (4–8 trilayers): the CDW transition temperature increases from 100 K^34^ in crystalline bulk to 135 K^35^.

Many theoretical and experimental efforts have been devoted to semiconducting TMDs, but only a few theoretical calculations have been performed on metallic few-layer TaSe_2_^36^,^37^. In this work, we have pursued detailed first-principles calculations to investigate the atomic structures, electronic and vibrational properties of few-layer TaSe_2_ in the undistorted normal states. Our calculations reveal a strong dependence of the electronic and phonon properties on the layer number and on the the stacking geometry, and we discuss the implications of the effect of dimensionality on the CDW transition in this material.

## Results

### Monolayer TaSe_2_

**Figure 1:**
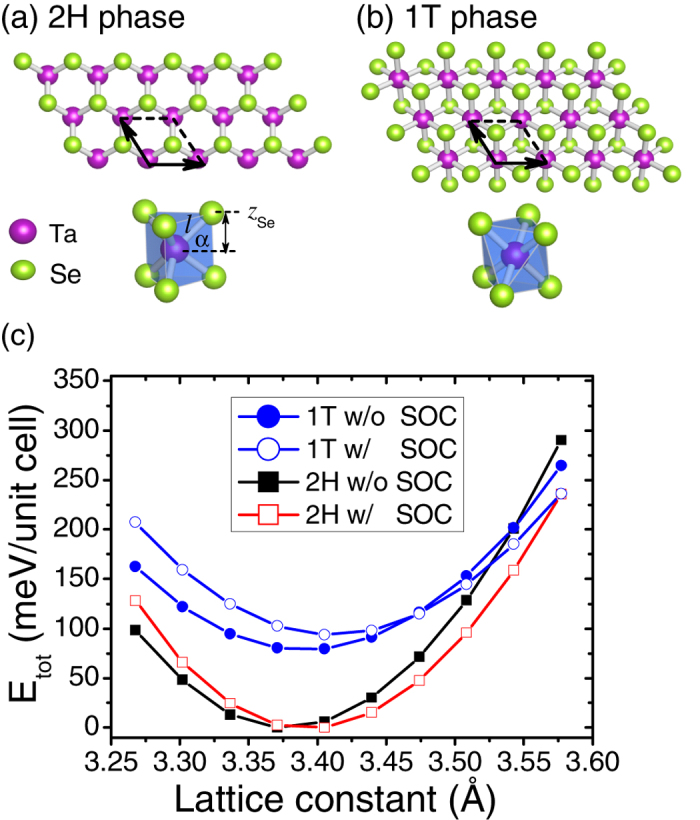
Crystal structures of monolayer TaSe_2_. (**a**) 2H phase and (**b**) 1T phase. The unit cells of the two phases have been indicated in (**a**,**b**), respectively. (**c**) Evolution of the total energy *E*_*tot*_ as a function of the lattice constant for monolayer TaSe_2_, with and without spin-orbit coupling. For clarity, *E*_*tot*_ are relative values with respect to the corresponding minima in each case.

The crystal structure of TaSe_2_ consists of Se-Ta-Se trilayer building block^7^. Depending on the relative rotation of the two Se layers within the trilayer unit, the bulk TaSe_2_ has two different phases: 2H and 1T^7^. In the 2H phase, the two Se layers form trigonal prismatic coordination, while they follow octahedral prismatic coordination in the 1T phase, as schematically shown in Fig. 1. The trilayer Se-Ta-Se structure in the 2H monolayer can be regarded as ABA stacking, while it changes to ABC stacking in the 1T phase^38^. As will be shown below, such a small structural variation results in a dramatic difference on the electronic and vibrational properties.

We start with monolayer TaSe_2_. Figure 1(c) depicts the total energy *E*_*tot*_ per unit cell as a function of the lattice constant *a*_0_ for both 2H and 1T phases. The atomic structures of 2H and 1T monolayers are schematically shown in Fig. 1(a,b), respectively. Figure 1(c) compares the energies with and without the spin-orbit coupling (SOC), and shows that the 2H phase is energetically more favorable than the 1T phase. Specifically, the *E*_*tot*_ in the 2H phase is about ~80 meV/unit cell lower than that in the 1T phase. Without SOC, the equilibrium lattice constant of 2H phase (3.378 Å) is 0.4% smaller than that of the 1T phase (3.391 Å). Our results are smaller than the experimental value 3.43 Å^38^ for 2H-TaSe_2_, but close to previous theoretical values (3.39 Å for 2H and 3.41 Å for 1T) with similar exchange-correlation functional^36^,^37^. This is understandable since LDA usually underestimates the lattice parameter by 1–2%. Note that the inclusion of SOC slightly increases the lattice constant in both cases. All these data along with a few other geometrical parameters have been listed in Table 1.

**Figure 2:**
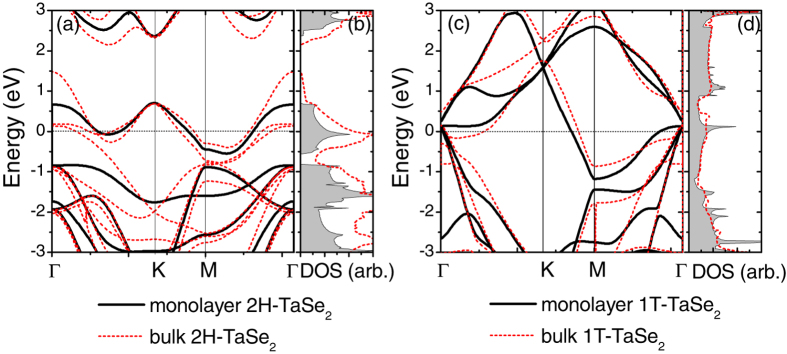
(**a**) Electronic band structure and (**b**) the density of states (DOSs) for monolayer 2H-TaSe_2_. (**c**,**d**) are results for monolayer 1T-TaSe_2_. Band structures and DOSs of bulk are shown as red dashed lines. The Fermi level has been shifted to zero.

In Fig. 2, we show the electronic band structures together with the density of states (DOSs) for monolayer TaSe_2_. Although the crystal structures of 2H and 1T phases are similar, the electronic band dispersions exhibit distinct features, as shown in Fig. 2(a,c), respectively. In the 2H phase, there is a separate narrow electronic band near the Fermi level, with width ~1.3 eV. This band originates from the 5*d* orbitals of the Ta atom under the crystal field formed by the six nearest-neighbor Se atoms in the octahedra prismatic coordination. In contrast, no such narrow band can be seen in the 1T case, leading to a distinct band dispersion near the Fermi level. This feature can be identified even more clearly from the DOSs, as shown in Fig. 2(b,d), respectively.

The band dispersions of bulk 2H-TaSe_2_ and 1T-TaSe_2_ are also presented in Fig. 2 for comparison. In bulk 2H-TaSe_2_, the unit cell has two layers of 2H monolayers stacked along the *z* direction. Accordingly, more bands show up near the Fermi level. As shown in Fig. 2(a), the conduction band minimum (CBM) of monolayer at Γ falls between the lowest and the second lowest conduction bands at Γ in the bulk. The band width in monolayer 2H-TaSe_2_ is also much narrower than in the bulk. In contrast, the band structure of monolayer 1T-TaSe_2_ is drastically different from its bulk counterpart. In particular, a sharp peak appears at the Fermi level in monolayer 1T-TaSe_2_, as shown in Fig. 2(d). Upon reducing the dimension from 3D to 2D, electrons in monolayer TaSe_2_ are confined into the nanosheet plane, offering a genuine 2D character to the electronic structure. One can expect that the reduced dimension alters the Fermi surface topology (as will be discussed below), implying possible dimensionality effects on their CDW transitions.

**Figure 3:**
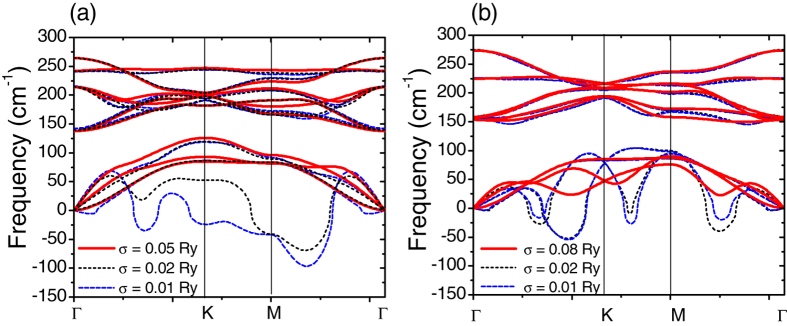


The phonon dispersions of monolayer 2H and 1T phases are shown in Fig. 3 (see Fig. S1 in the Supplementary Materials for results with different pseudopotentials). There are three atoms in the unit cell, yielding nine phonon bands in total. Among them, three are acoustic branches and six are optical branches. In both phases, the acoustic branches are separated from the six optical branches, exhibiting an indirect phonon frequency gap of about 20 cm^−1^ for the 2H phase and 40 cm^−1^ for the 1T phase, respectively. From Fig. 3, one can identify that there is a significant portion of the longitudinal acoustic phonon band showing negative phonon frequencies (more than −50 cm^−1^) along the ΓK and ΓM directions for both 2H and 1T phases (data with smearing parameter *σ* = 0.01 Ry). This result shows that the undistorted monolayer TaSe_2_ is not mechanically stable in the ground state.

The parameter *σ*, which has been used in the electronic self-consistent calculations to accelerate the convergence, smears out the abrupt change of the Fermi-Dirac statistics in the ground state, and represents the electronic temperature. By changing this parameter, one can qualitatively assess the effect of temperature on the phonon properties of the system^36^ and possible Kohn anomaly^39^. Figure 3 shows the phonon dispersions calculated with three different values of *σ*. When *σ* → 0, the ground state will be recovered. Clearly, the phonon dispersions show big negative mode along the acoustic branch in both phases with *σ* = 0.01 Ry. While increasing *σ*, the negative phonon branches become less evident, and finally completely change to positive phonon modes with *σ* = 0.05 Ry in 2H phase and with *σ* = 0.08 Ry in 1T phase. The dependence of the phonon dispersions on the smearing temperature, in accord with the different transition temperatures in bulks, implies that similar CDW transitions might also exist in monolayer TaSe_2_: Upon cooling to low temperatures, crystalline monolayer TaSe_2_ undergoes a series of structural phase transitions, from normal state to incommensurate CDW phase and to commensurate CDW phase eventually. Note that the smearing parameter, which indicates the electronic temperature, should not be directly compared with the real ambient temperature of the crystal, which includes the lattice temperature as well.

Overall, our results of monolayer are in accord with available experimental observations for bulk 2H-TaSe_2_ and 1T-TaSe_2_. A strong anomaly has been reported on the longitudinal acoustic phonon branch near the wave vector *q* = Γ*M* 2/3 in both 2H and 1T phases^7^,^14^,^15^,^16^,^18^,^19^,^21^. Phonon frequencies at this anomaly show a remarkable softening as the temperature is decreased towards the transition temperature^7^,^14^,^15^,^16^,^18^,^19^,^21^. It has also been argued that there is a Kohn anomaly associated with the phonon mode at the Γ point in the 2H bulk^40^. However, our results of monolayer TaSe_2_ only show an additional dramatic softening close to half of Γ − *K* line in monolayer TaSe_2_. As will be discussed further below, the reduced dimension from bulk to monolayer dramatically alters the Fermi surface topology as well as the phonon band dispersions.

### Few-layer TaSe_2_

**Figure 4:**
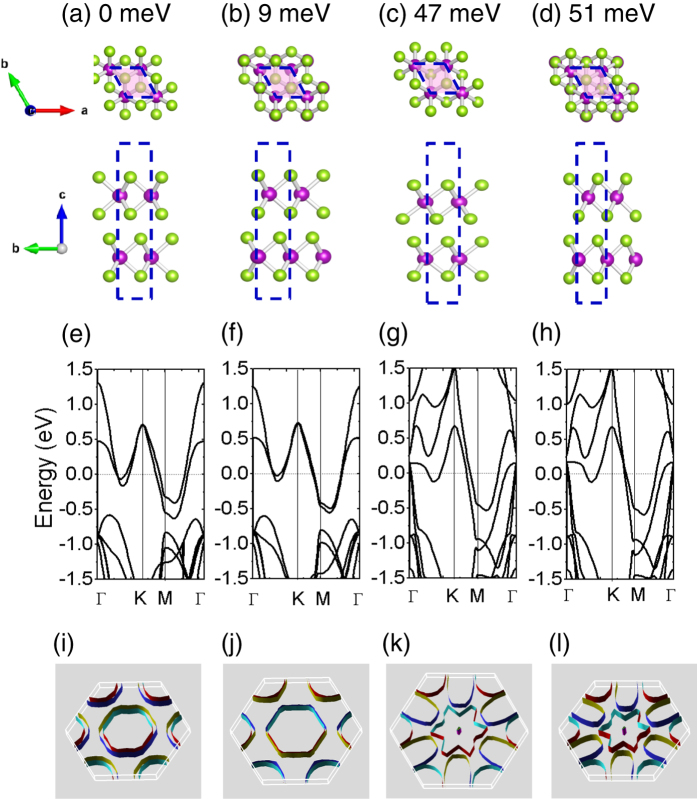
Stacking geometries and the corresponding band structures for various bilayer TaSe_2_. (**a**) Two H layers stacked together, with upper layer rotated 60° with respect to bottom layer. (**b**) Two H layers stacked together, with the upper layer translated along the Ta-Se bond direction. (**c**) One H layer stacked with a T layer. (**d**) One H layer stacked with a rotated T layer. The relative total energy per unit cell has been indicated for each configuration. (**e**–**h**) are the corresponding band structures without SOC. The Fermi level has been shifted to zero. (**i**–**l**) are the corresponding 2D Fermi surfaces.

Few-layer TaSe_2_ belongs to an intermediate design between bulk TaSe_2_ and monolayer sheet, so their band structures will be reminiscent of both. The van der Waals interlayer interaction, which gives rise to the band dispersions out of the 2D atomic plane in bulk, is now responsible for the band splitting/mixing between isolated monolayer bands occurring in few-layer TaSe_2_. One can expect that both of the hole and electron charge carriers may be present since different bands are present in the same energy range near the Fermi level (see Fig. 4 for example). The number of layers and the geometry dependence of the interlayer interaction are therefore key parameters influencing the transport properties of few-layer TaSe_2_, which may be important for their interconnect applications.

We discuss bilayer TaSe_2_ first. The van der Waals interlayer interaction permits the facile mechanical delamination and exfoliation. Several TaSe_2_ polytypes that differ in the relative orientation of the layers and stacking arrangements could form when the thicknesses are down to few layers^38^. We have considered 12 different configurations, with H and/or T layers stacking together with various shifting and/or rotations. Among all studied configurations, the energy of bilayer 1T-TaSe_2_ (denoted as 1T-2L) is about 136 meV/unit cell higher than the bilayer 2H-TaSe_2_ (denoted as 2H-2L), which is the lowest configuration. Figure 4 shows the four stacking geometries with their relative total energies per unit cell calculated based on LDA. We find that the configuration with the stacking geometry following the bulk 2H-TaSe_2_ has the lowest energy (set to be the reference point 0, Fig. 4(a)). Other than that, the second lowest structure, as shown in Fig. 4(b), is the double H layers stacked with a relative translational shift, which is only 9 meV/unit cell higher than the configuration in (a). Note that this energy is already within the room temperature range. The combinations of H and T layers, on the other hand, are relatively higher in energy, but still lower than the double T layer structure (136 meV). To verify the relative energies for these four configurations, we have further calculated the total energy for each configuration with van der Waals corrections at the vdW-DF2 level^41^,^42^,^43^ using VASP^44^,^45^. After further relaxations of the structures listed in Fig. 4(a–d), the calculated relative energies are 0, 2.2 meV, 22 meV and 23 meV, respectively. The results are in the same order as LDA predicts. Since the energy difference between these configurations are far lower than the 1T bilayer case, whose bulk form exists at high temperatures, we infer that down to few-layer TaSe_2_, various stacking geometries could be possible. Depending on the fabrication methods and processes, a mixture of various polytypes in few-layer TaSe_2_ might be present. This result supports recent experimental observations: the possible existence of various polytypes during the fabrication of few-layer TaSe_2_^26^.

The corresponding band structures for the four bilayer configurations are shown in Fig. 4(e–h), respectively. The configurations stacked with two H layers have similar band dispersions, as shown in Fig. 4(e,f). These band dispersions are distinct from those of configurations consisting of H and T layers (4(g) and 4(h)). The latter two are similar to the combined band structures of H and T monolayers. These results show that the electronic band dispersions are significantly dependent on the stacking geometry in bilayer TaSe_2_.

The strong stacking-dependent electronic properties are manifested more clearly from the 2D Fermi surfaces as shown in Fig. 4(i–l), which are plotted using XCrysDen software^46^. For 2D crystals, the Fermi surface reduces to contour lines in the first BZ plane. In a 3D plot, the 2D Fermi surfaces consist of cylindrical sheets that extend perpendicular to the 2D BZ plane. In Fig. 4(i–l), the Γ-centered Fermi surfaces show two important features. First, for bilayers consisting two H layers, the Fermi surfaces have a shape similar to each other, as shown in Fig. 4(i,j), respectively. Only the splittings between neighboring surface sheets are slightly different. This is also true for the bilayers consisting of H and T layers, as shown in Fig. 4(k,l). Therefore, the shape of Fermi surface is mainly determined by the types of layers stacked together. Secondly, we find that the Fermi surfaces are dramatically altered in Fig. 4(k,l) when H and T layers are mixed together. The star-shaped Fermi surface sheets are interesting and distinct from both H and T phases (as will be shown later), indicating the possibilities of tuning the Fermi surface topology by fabricating van der Waals heterostructures using H and T layers.

**Figure 5:**
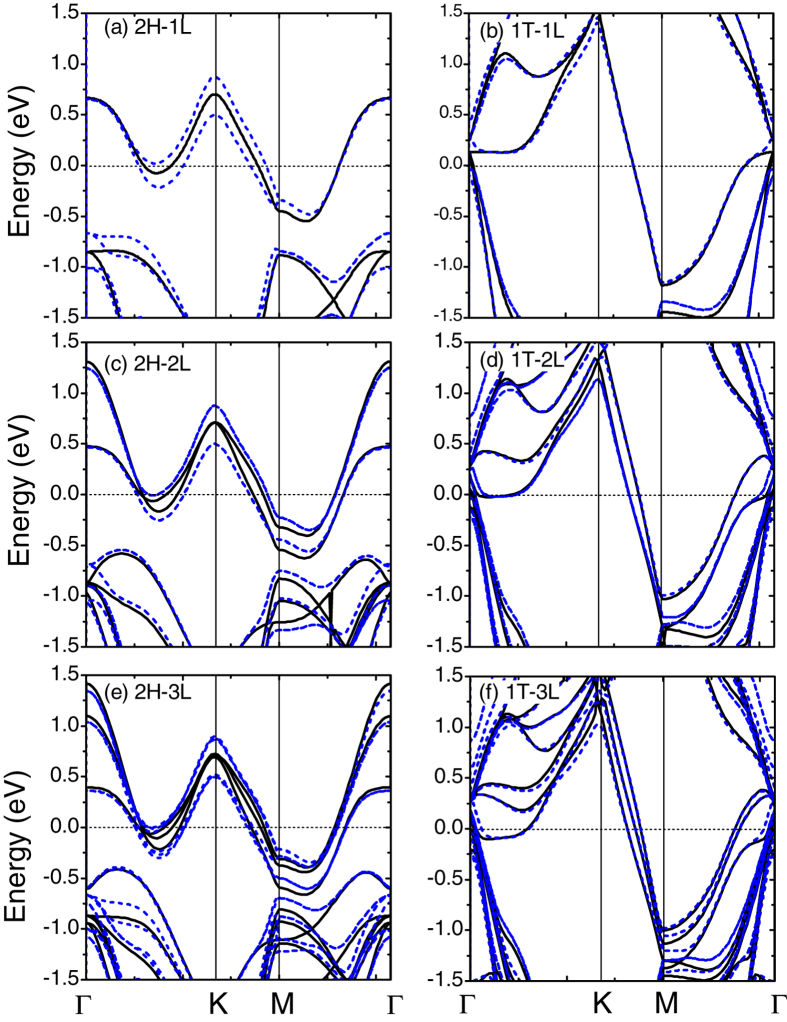
Band dispersions for few-layer 2H-TaSe_2_ and 1T-TaSe_2_ as a function of layer number. Black solid (blue dashed) lines are results without (with) SOC. The Fermi level has been shifted to zero.

Using the H and T layers as building blocks, we also calculated the band structures for few-layer 2H-TaSe_2_ and 1T-TaSe_2_ up to three trilayers. The calculated structural parameters for few-layer TaSe_2_ have been summarized in Table 1. For the 2H case, the stackings follow the 2H bulk sequence, as shown in Fig. 4(a). For the T phase, TaSe_2_ trilayers follow AA stacking as in bulk 1T-TaSe_2_. As shown in Fig. 5, the band structures are strongly dependent on the layer number.

In Fig. 5, we also show the band dispersions with an explicit inclusion of SOC. The band dispersions in the 1T phase are similar to those without SOC, while more evident splittings occur on the bands of few-layer 2H-TaSe_2_ (see Fig. 5(a, c, e)), leading to more delicate change on the bands near the Fermi level. Thus, SOC changes the Fermi surface topology, particularly in the 2H phase^27^. Another interesting observation is that in the 1T few-layer TaSe_2_, the bands along *KM* line seem to follow a linear band dispersion, as can be seen in Fig. 5(b, d, f).

**Figure 6:**
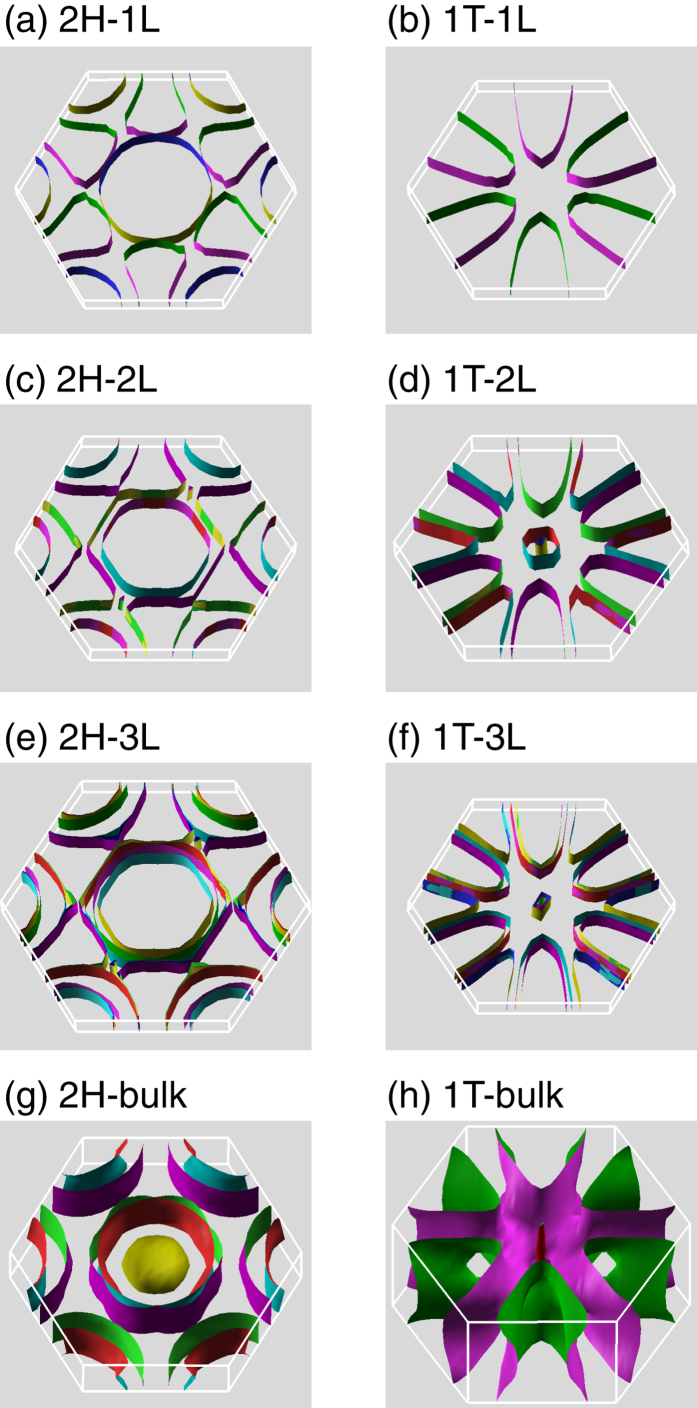


The evolutions of the Fermi surface topology as a function of layer number for both 2H and 1T phases are presented in Fig. 6, with SOC explicitly included. Overall, the Fermi surfaces of few-layer TaSe_2_ mimic their bulk. There is, however, a sharp change on the Fermi surface from monolayer to bilayer for both 2H and 1T phases. Specifically, the Fermi contours centered at *M* point evolve from 2H-1L (Fig. 6(a)) to 2H-2L (Fig. 6(c)), which begins to center at *K* point. From 1T-1L to 1T-2L, additional Fermi surface sheets show up at the BZ corner (*K* point), as depicted in Fig. 6(b,d). In addition, small Fermi surface sheets appear at around Γ in 1T-2L. Clearly, with reduced dimensions, the Fermi surface shows distinct topology from that in the bulk and thus is sensitive to the layer number as well as stacking geometry.

**Figure 7:**
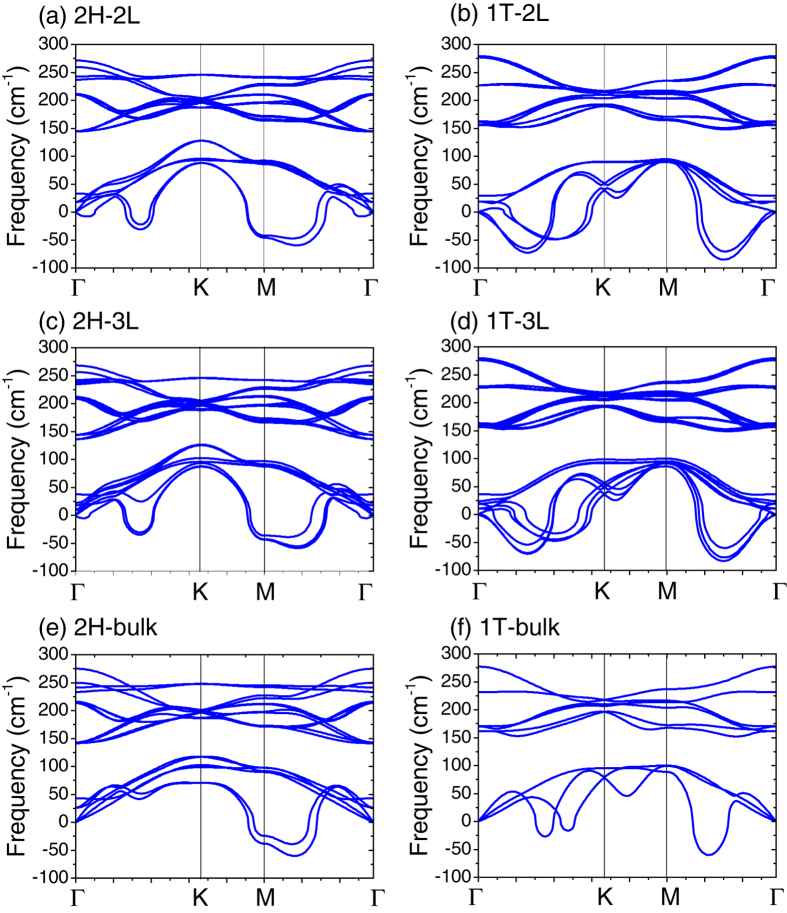
Phonon dispersions of few-layer 2H-TaSe_2_ and 1T-TaSe_2_ as a function of layer number. The phonon bands of bulk 2H-TaSe_2_ and 1T-TaSe_2_ along the high symmetry path on the *q*_*z*_ = 0 plane are also shown for comparison.

The dimensionality effects are also evident on the phonon properties of few-layer TaSe_2_. Figure 7 illustrates the phonon dispersions as a function of layer number for both 2H and 1T phases. The results of bulk are also shown for comparison. All calculations used the same smearing of *σ* = 0.02 Ry. Due to the interlayer coupling, there are small splittings on the optical phonon branches. Additional low-frequency phonon branches (below 50 cm^−1^) can be seen for bilayer and trilayer TaSe_2_. These modes correspond to breathing and shearing modes, which will be discussed further below. Most importantly, there are interesting evolutions on the acoustic branches which exhibit negative phonon modes, as shown in Fig. 7. In bulk 2H-TaSe_2_, only the Γ*M* line shows negative phonon branches (Fig. 7(e)). In contrast, there are additional negative phonon branches along the Γ*K* line for both 2H-2L and 2H-3L. This result has not been reported before, and possibly indicates different structural phase transitions in few-layer TaSe_2_ as compared with their bulk form. In the case of 1T phase, the negative phonon branches along the Γ*K* line are less evident than the Γ*M* line (Fig. 7(f)). Upon reducing from bulk to 2L, these negative branches are greatly enhanced and become comparable in magnitude with those along the Γ*M* direction. These results highlight the dimensionality effects on the phonon properties in few-layer TaSe_2_.

Finally, it is instructive to compare the interlayer interactions in few-layer TaSe_2_ with other layered 2D systems. For bilayer TaSe_2_, the binding energy can be evaluated by *E*_*b*_ = −(*E*_*tot*_ − *LE*_*mono*_), with the layer number *L* = 2. *E*_*tot*_ (*E*_*mono*_) is the total energy for *L*-layer (monolayer) TaSe_2_. The calculated *E*_*b*_ are 190 meV and 170 meV for the 1T and 2H phases, respectively. Interestingly, the *E*_*b*_ in 1T is about 20 meV higher than in 2H. Note that the binding energy in this metallic TMD is about two times stronger than that calculated for semiconducting MoS_2_ (81 meV for bilayer MoS_2_), and also three times larger than in bilayer graphene (48 meV). Surprisingly, our further calculations^44^,^45^,^47^ with van der Waals corrections at the vdW-DF2 level^41^,^42^,^43^ yield almost the same binding energy for all these materials: ~19 meV/Å^2^, implying the same van der Waals nature in all of these layered crystals.

### Vibrational properties of few-layer TaSe_2_

In this section, we discuss the vibrational modes at the Brillouin zone center Γ. Some modes are Raman active and can be probed nondestructively by Raman spectroscopy. These Raman-active modes provide useful clues to the structural and electronic changes during the CDW transitions. Studies of the temperature dependence of the Raman spectra near the CDW phase transitions are particularly useful for understanding the dynamics of transitions to the CDW state^14^,^15^.

**Figure 8:**
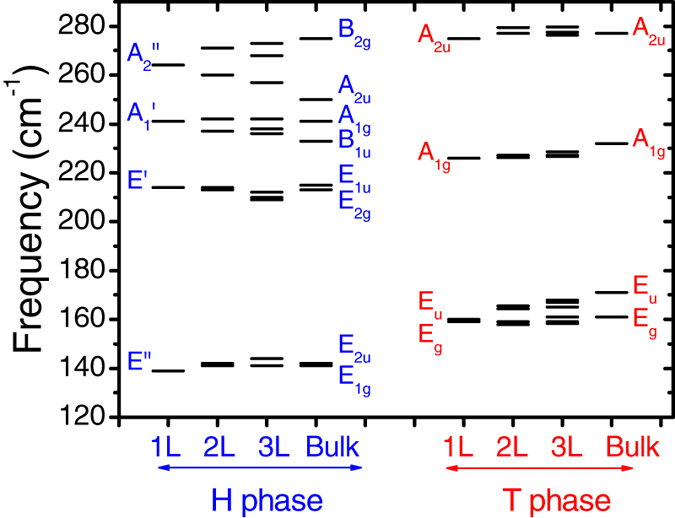
Phonon frequencies for the high-energy optical modes at Γ in few-layer TaSe_2_. Symmetries for typical modes are labeled explicitly. The 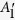, *E*′, *A*_1*g*_ and *E*_*g*_ are Raman active.

Figure 8 shows the evolution of the high-frequency vibrational mode frequencies as a function of layer number for both 2H and 1T phases. The vibrational modes for the bulk are also shown. In Fig. 8, the symmetries for the modes in monolayer and bulk are explicitly indicated. The monolayer 2H-TaSe_2_ belongs to point group of *D*_3*h*_. The Γ phonon modes can be represented by 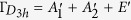 + *A*_1_″ + *A*_2_″ + *E*″. The 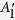 mode at about 241 cm^−1^, the *E*′ mode at 214 cm^−1^, and the *E*″ mode at 140 cm^−1^ are all Raman-active. These modes correspond to the characteristic *A*_1*g*_, *E*_2*g*_ and *E*_1*g*_ modes in bulk 2H-TaSe_2_ (with *D*_6*h*_ symmetry)^15^. In few-layer 2H-TaSe_2_, recent experiments already confirmed the Raman peaks at around 235 cm^−1^, 208 cm^−1^, and 150 cm^−1^ 26. Our results are also in good agreement with the Raman observation by Yan *et al.*^3^. They found that the *E*_2*g*_ mode falls into the range of 207–210 cm^−1^, while the *A*_1*g*_ mode is in the range of 233–235 cm^−1^.

In contrast, the monolayer 1T-TaSe_2_ possesses *D*_3*d*_ point group symmetry. The phonon modes at Γ can be decomposed as 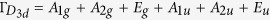. The two Raman-active modes are *A*_1*g*_ at 226 cm^−1^ and *E*_*g*_ mode at 159 cm^−1^, respectively, as shown in Fig. 8. Unlike the case of 2H-TaSe_2_, the calculated phonon vibrational frequencies differ substantially from the Raman data^15^. As temperature decreases, the high-lying *A*_1*g*_ mode at 190 cm^−1^ was shown to be almost constant experimentally, while the low-lying *A*_1*g*_ mode at 99 cm^−1^ disappeared upon the transition to incommensurate and commensurate CDW state^15^. A more recent Raman measurement^48^ yielded 187 and 177 cm^−1^ for the *A*_1*g*_ and *E*_*g*_ modes, respectively. These values are different from our calculations for the undistorted structure. In addition, it has been reported that the low-lying *E*_*g*_ mode at 80 cm^−1^ is almost independent of the temperature change^15^. However, the *E*_*g*_ mode in the normal state here is about 159 cm^−1^. The reason of such a big discrepancy is not clear yet. More accurate experimental data about the structural change during the CDW transition will be helpful to resolve this discrepancy.

**Figure 9:**
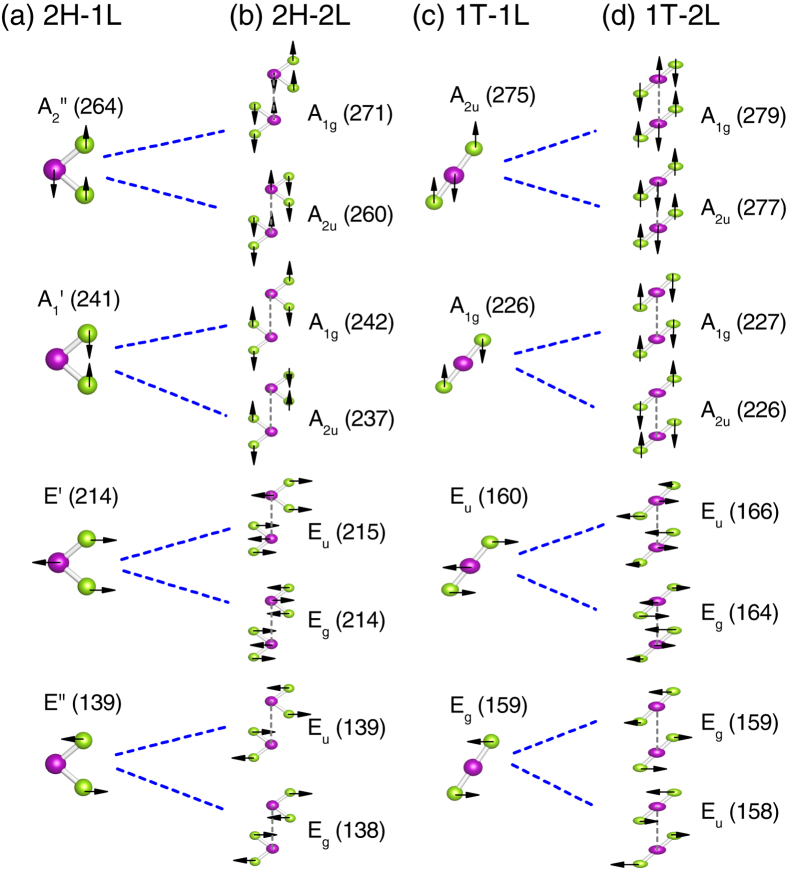
Phonon eigenvectors for the modes at *q* = Γ in TaSe_2_. (**a**) Monolayer 2H-TaSe_2_; (**b**) bilayer 2H-TaSe_2_; (**c**) monolayer 1T-TaSe_2_; (**d**) bilayer 1T-TaSe_2_. Dashed lines show the mode evolutions from monolayer to bilayer in both H and T phases, respectively. Symmetry and frequency (in cm^−1^) of each mode have been indicated. The *E*′, *A*′, *E*_*g*_, *A*_1*g*_ modes are Raman active.

In few-layer TaSe_2_, interlayer interactions lead to mode splittings. This has been discussed in detail for semiconducting TMDs^49^. As shown in Fig. 8, these splittings are also evident in TaSe_2_. For example, the *E*″ mode in monolayer 2H-TaSe_2_ splits into two branches: *E*_2*u*_ and *E*_1*g*_ in the bulk. The latter becomes Raman active. In Fig. 9, the evolutions of the vibrational patterns from monolayer to bilayer have been depicted in more detail for both 2H and 1T phases. Overall, the interlayer interactions have small effects on the phonon mode splittings (within 2 cm^−1^). This is in contrast with the electronic band splittings as shown in Fig. 5. The electronic band structures near the Fermi level are more sensitive to the interlayer interactions as compared with the phonon modes.

Bulk 1T-TaSe_2_ has AA-type stacking of the trilayer unit, with the symmetry of *D*_3*d*_ (see Table 1). Therefore, the optical phonon modes at Γ consist two non-degenerate out-of-plane modes: *A*_1*g*_ and *A*_2*u*_, and two doubly degenerate in-plane modes: *E*_*u*_ and *E*_*g*_. In the *A*_1*g*_ mode, Ta layer does not move, while the two Se layers vibrate out of phase. In the *A*_2*u*_ mode, the two Se layers vibrate in phase, but vibrate out of phase with respect to the Ta layer, as shown in Fig. 9(c). On the other hand, the symmetry of the 2H monolayer is *D*_3*h*_, which has *A*_2″_ and 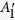 out-of-plane modes, and two doubly degenerate *E*′ and *E*″ in-plane modes. The Raman active modes 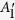 has an out-of-phase motion between two Se layers, with Ta layer fixed. Another Raman-active mode *E*′ is an in-plane mode. All of these modes have been schematically shown in Fig. 9.

The state-of-the-art Raman setup offers access to frequencies down to 10 cm^−1^ and spectral resolution of 0.5 cm^−1^, powerful to probe the low-frequency modes that are directly associated with interlayer coupling^50^,^51^. Recent studies have revealed that interlayer couplings in van der Waals layered materials such as few-layer graphene and TMDs can create a set of layer-shearing modes (LSMs) and layer-breathing modes (LBMs) that involve lateral and vertical displacement of individual layers, respectively^50^,^51^. In LSM and LBM, the layers vibrate relatively as a whole with respect to each other. These interlayer phonon modes provide sensitive probes to layer thickness, stacking order, and surface adsorbates of 2D materials^50^.

**Figure 10:**
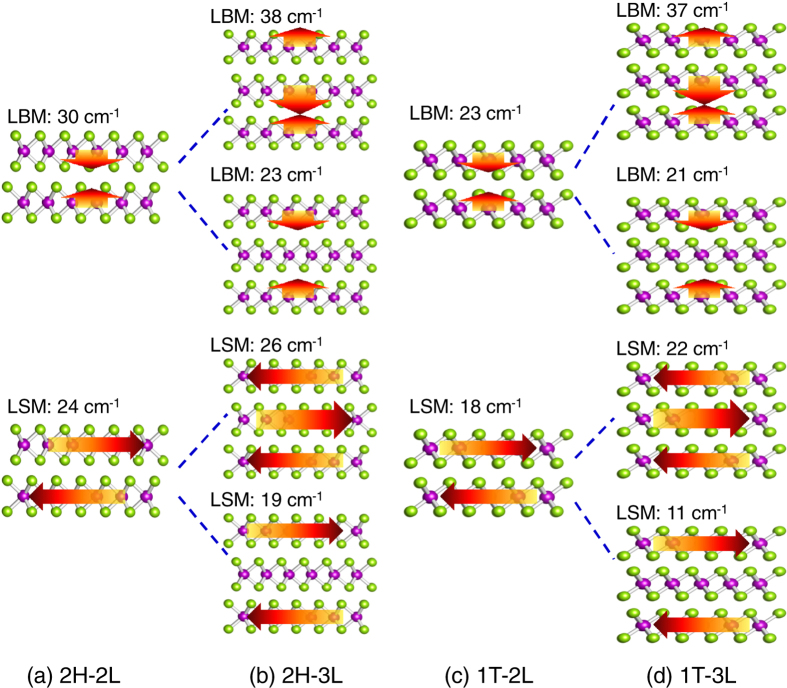
The low-frequency layer-shearing modes (LSMs) and layer-breathing modes (LBMs) in bilayer and trilayer TaSe_2_. Arrows indicate the relative displacements of each layer.

We have also analyzed the low-frequency modes in bilayer and trilayer 2H-TaSe_2_ and 1T-TaSe_2_. As shown in Fig. 10, the LSM in 2H-2L is 24 cm^−1^, slightly smaller than the LSM mode (26 cm^−1^) in bulk 2H-TaSe_2_ (not shown). On the other hand, the LBM of 2H-2L is at 30 cm^−1^, much smaller than the LBM of 43 cm^−1^ in bulk. The frequencies of these modes are in the same range of the reported low-frequency modes in Bernal 2L-MoS_2_ and 2L-WSe_2_, in which the interlayer shearing modes fall into 17–22 cm^−1^ while LBMs fall in 30–40 cm^−1^ 50. All the LBM and LSM modes in 2H-2L and 1T-2L are Raman active, and therefore could provide a useful fingerprint to identify these two phases.

For more than two layers, both LSM and LBM will split because of interlayer coupling. As shown clearly in Fig. 10, the splitting of modes is large: about 8–10 cm^−1^ for LSM and 15–16 cm^−1^ for LBMs. The findings here should be useful to identify the layer stackings and layer numbers in few-layer TaSe_2_ based on the Raman detection of the LSMs and LBMs.

## Discussion

In summary, we have studied the structural, electronic and phonon properties of few-layer TaSe_2_. Our results show that various polytypes consisting of H and T layers are energetically possible for few-layer TaSe_2_, which is dependent on the growth and fabrication processes. The electronic structure and phonon vibrations are found to be strongly dependent on the layer number as well as the stacking geometry. The evolution of the Fermi surface and phonon dispersions with respect to layer number suggests interesting and different structural phase transitions in few-layer TaSe_2_, highlighting the dimensionality effects in this CDW material. The detailed analysis of the vibrational modes provides a basis for the Raman spectra of this metallic TMD.

## Methods

Calculations were carried out using density-functional theory (DFT)^52^,^53^ as implemented in the Quantum ESPRESSO code^54^ with the Perdew-Wang (PW) exchange-correlation functional. According to our test calculations, the local density approximation (LDA) exchange-correlation functional is relatively better than the general-gradient approximation (GGA) in describing the vibrational properties and the layer-layer interactions. This is similar to the semiconducting TMDs as reported in ref. 49. For van der Waals materials, an accurate description of interlayer coupling may require corrected exchange functionals as proposed in refs 55,56. However, our calculations showed that LDA already predicted vibrational phonon frequencies close to experimental data (especially in the 2H phase). Therefore, we reported all the results within LDA. Norm-conserving pseudopotentials were employed for the description of interactions between core and valence electrons. The cutoff energy in the plane wave expansion was set to 50 Ry. A Monkhorst-Pack uniform *k*-grid of 36 × 36 × 1 was employed. A Methfessel-Paxton smearing^57^ of 0.02 Ry was adopted in all calculations. A vacuum region of 20 Å was introduced along the out-of-plane (*z*) direction to eliminate spurious interactions among periodic images. For phonon calculations, we used a 6 × 6 × 1 uniform *q*-grid. Since both Ta and Se are heavy atoms, calculations with an explicit inclusion of the relativistic spin-orbit coupling (SOC) have also been performed for comparison, using a fully relativistic Perdew-Zunger PAW pseudopotentials. All the atomic structures and unit cells have been fully relaxed until the forces on atoms are smaller than 0.02 eV/Å. Additional calculations with the van der Waals (vdW) corrections were performed using VASP^44^,^45^. The PBE functional^47^ with vdW corrections at the vdW-DF2 level^41^,^42^,^43^ was employed.

## Additional Information

**How to cite this article**: Yan, J.-A. *et al.* Structural, electronic and vibrational properties of few-layer 2H- and 1T-TaSe_2_. *Sci. Rep.* **5**, 16646; doi: 10.1038/srep16646 (2015).

## Supplementary Material

Supplementary Information

## Figures and Tables

**Table 1:** Calculated structural parameters for few-layer 2H-TaSe_2_ and 1T-TaSe_2_, respectively.

	2H-TaSe_2_				1T-TaSe_2_			
	1L	2L	3L	bulk	1L	2L	3L	bulk
Symmetry	D_3*h*_	D_3*d*_	D_3*h*_	D_6*h*_	D_3*d*_	D_3*d*_	D_3*d*_	D_3*d*_
*a*_0_ (Å)	3.378	3.378	3.378	3.375	3.391	3.384	3.384	3.384
*c* (Å)	—	6.106	6.112	6.140	—	6.123	6.130	6.137
*z*_Se_ (Å)	1.649	1.640	1.640	1.651	1.650	1.637	1.636	1.644
*l*_Ta−Se_ (Å)	2.554	2.549	2.549	2.554	2.556	2.549	2.549	2.553
*α*	40.2°	40.1°	40.1°	40.3°	39.9°	40.0°	39.9°	40.1°

Here *a*_0_, *c*, and *z*_*Se*_ are the in-plane lattice constant, the vertical distance between two neighboring Ta layers, the vertical *z* coordinate of Se atoms relative to the Ta plane, respectively. *l*_*Ta*−*Se*_ is the bond length between Ta and Se atoms, and *α* is its angle with respect to the Ta plane, as schematically shown in Fig. 1.
