# Supplementary material for: Structural, electronic and vibrational properties of few-layer 2H- and 1T-TaSe2
Source: Sci Rep. 2015 Nov 16;5:16646. doi: 10.1038/srep16646 (PMC4644971; doi:10.1038/srep16646)
Supplement: Supplementary Information [file srep16646-s1.docx]

**Supplementary Materials**

**Structural, electronic and vibrational properties of**

**few-layer 2H- and 1T-TaSe_2_**

**Jia-An Yan,^1^ Mack A. Dela Cruz,^1^ Brandon Cook,^2^ and Kalman Varga^3^**

^1^Department of Physics, Astronomy, and Geosciences, Towson University, 8000 York Road, Towson, Md 21252, USA

^2^Center for Nanophase Materials Sciences, Oak Ridge National Laboratory, P.O. Box 2008, Oak Ridge, TN, 37831 USA

^3^Department of Physics and Astronomy, Vanderbilt University, Nashville, Tennessee 37235, USA

1. **Phonon dispersions of monolayer TaSe_2_.**

The phonon dispersions of monolayer TaSe_2_ slightly depends on the pseudopotentials (PPs) employed in the calculations. We have performed additional calculations using norm-conserving (NC) and ultrasoft PPs with Perdew-Zunger (PZ) LDA exchange-correlation functional. The cutoff energies are set to 50 and 35 Ry for NCPP and USPP, respectively. The smearing parameter σ is set to 0.02 Ry. In the case of USPP, the core corrections are also included and the charge density cutoff energy is 280 Ry. A 6×6×1 q-grid has been used. The results are shown in Fig. S1. Overall, they are similar to that obtained using PW NCPP in the manuscript. USPP yields slightly different negative acoustic branch as compared with NCPP, especially in 2H-1L TaSe_2_.


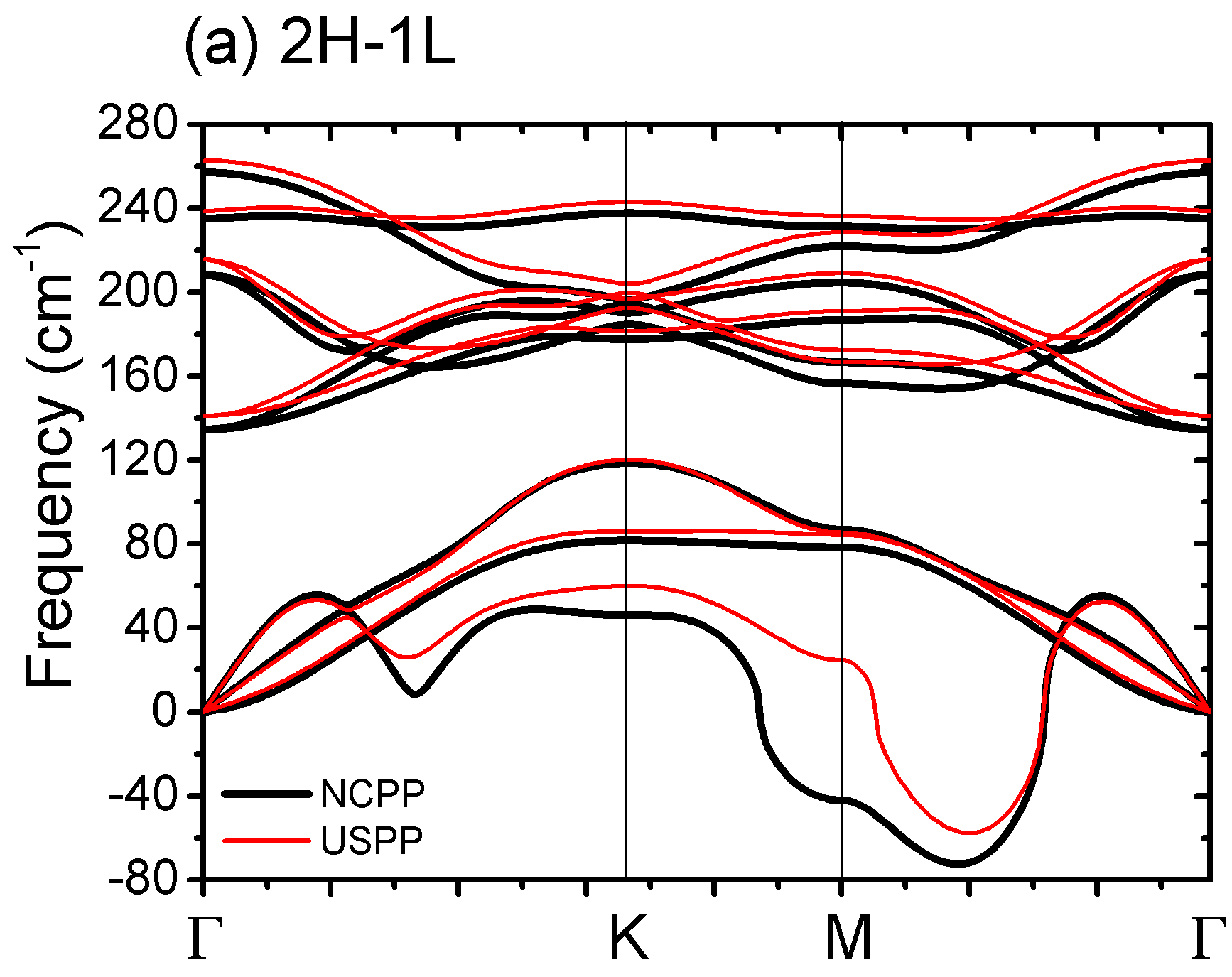

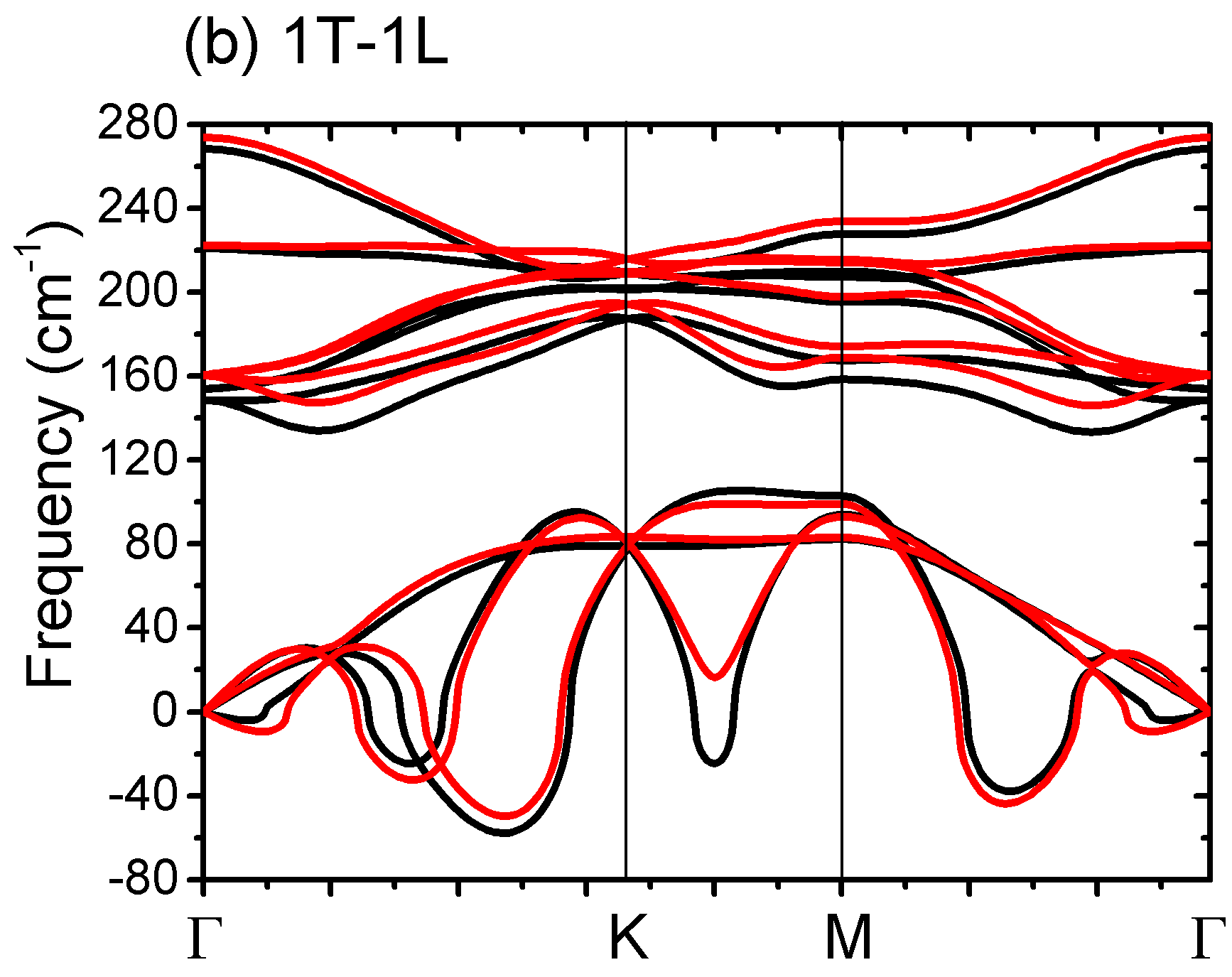


Fig. S1. Phonon dispersions of monolayer (a) 2H-TaSe_2_ and (b) 1T-TaSe_2_ calculated using norm-conserving (black) and ultrasoft (red) pseudopotentials. The Perdew-Zunger exchange-correlation functional is adopted.
